# New horizons in sexual health: exploring PrEP and HIV incidence in adolescents

**DOI:** 10.11606/s1518-8787.2024058supl1ap

**Published:** 2024-10-11

**Authors:** Alexandre Grangeiro, Eliane Miura Zucchi, Laio Magno, Unai Tupinambás, Dirceu Bartolomeu Greco, Inês Dourado

**Affiliations:** I Universidade de São Paulo. Faculdade de Medicina. São Paulo, SP, Brasil Universidade de São Paulo Faculdade de Medicina SP São Paulo Brasil; II Universidade Católica de Santos. Programa de Pós-Graduação em Saúde Coletiva. Santos, SP, Brasil Universidade Católica de Santos Programa de Pós-Graduação em Saúde Coletiva SP Santos Brasil; III Universidade do Estado da Bahia. Departamento de Ciências da Vida. Salvador, BA, Brasil Universidade do Estado da Bahia Departamento de Ciências da Vida BA Salvador Brasil; IV Universidade Federal de Minas Gerais. Faculdade de Medicina. Belo Horizonte, MG, Brasil Universidade Federal de Minas Gerais Faculdade de Medicina MG Belo Horizonte Brasil; V Universidade Federal da Bahia. Instituto de Saúde Coletiva. Salvador, BA, Brasil Universidade Federal da Bahia Instituto de Saúde Coletiva BA Salvador Brasil

## APRESENTAÇÃO

A meta da Organização das Nações Unidas (ONU) de eliminar o HIV como problema de saúde pública, até 2030, é factível do ponto de vista teórico e científico ^1^ . Com efeito, diferentes estudos têm mostrado que o binômio tratamento oportuno de pessoas vivendo com HIV e a prevenção de novos casos de infecção, com as tecnologias de saúde disponíveis, é altamente eficaz para reduzir significativamente a incidência e mortalidade por HIV ^2^ . Entretanto, o alcance dessa meta tem enfrentado grandes desafios, como as inequidades, o estigma, o financiamento insuficiente, os déficits na estrutura de serviços e na articulação intersetorial e um relativo baixo engajamento de comunidades e dos formuladores de políticas ^1^^,^^3^^–^^6^ .

Diante disso, o Programa das Nações Unidas para Aids (Unaids) tem chamado atenção para o fato de que os países que mais avançaram na eliminação do HIV foram aqueles que promoveram informação, focalização das ações com maior impacto na epidemia, mobilização e participação social, além da devida articulação da resposta e de seus atores ^1^ . Isso pode ser observado em diferentes contextos, como em países africanos fortemente atingidos pela epidemia ^7^ e naqueles com maior renda ^8^ .

Em contrapartida, o perfil da epidemia de HIV no Brasil tem mudado significativamente, com um cenário que conjuga uma nova onda de aumento da incidência ^9^^,^^10^ , a consolidação de subepidemias regionais, marcadas pelas desigualdades sociais ^11^^–^^13^ e uma crescente incidência entre adolescentes e adultos jovens, especialmente entre homens cisgêneros que fazem sexo com homens, travestis e mulheres transexuais ^9^^,^^14^ .

O aumento da epidemia em adolescentes pode ser explicado por uma combinação de fatores ^14^ relacionados ao ciclo de vida, como a experimentação de práticas sexuais e a necessidade de lidar, desde cedo, com o estigma relacionado à orientação sexual e à identidade de gênero; mas, também, a uma mudança geracional marcada pela percepção de menor gravidade do HIV e a novas formas de exercer a sexualidade. Isso tem levado a uma espécie de ciclo vicioso, com o aumento da frequência de iniciação sexual sem o uso de preservativos e o sexo sem proteção ^15^ , especialmente em adolescentes com maior vulnerabilidade social ^16^ .

Uma das oportunidades para reverter esse cenário tem sido a Profilaxia Pré-exposição Sexual (PrEP) que, conjugada a outros métodos preventivos, se constitui como uma opção altamente efetiva ^17^ para populações em maior risco de infecção, especialmente aqueles que não querem ou não conseguem utilizar o preservativo de forma consistente e duradora. O aumento das taxas populacionais de uso da PrEP está associado ao decréscimo da incidência ^4^^,^^8^^,^^18^ e isso tende a ser ainda mais acentuado com a introdução de novos esquemas profiláticos de longa duração, que conferem maior comodidade e prometem reduzir a não adesão e a interrupção do uso ^2^ . Exemplos nesse sentido são os resultados de um ensaio clínico denominado de Purpose 1, que foi realizado com mais de 2 mil mulheres cisgênero, utilizou um medicamento injetável aplicado semestralmente e não registrou nenhuma infecção por HIV ao longo do estudo ^19^ .

Apesar desses resultados promissores, o uso de PrEP tem sido marcado por inequidades no acesso, pois concentra-se, fundamentalmente, em populações e regiões com maior nível socioeconômico ^2^^,^^6^^,^^20^ . No Brasil, por exemplo, as populações mais vulneráveis socialmente são as mais afetadas por essa desigualdade; tanto que mulheres cis, incluindo profissionais do sexo, têm chance 10 vezes menor de usar PrEP do que homens cis. Essa diferença é duas vezes maior entre negros e brancos; e entre aqueles com idade de 18 a 24 e de 30 a 39 anos ^21^ . Essas inequidades têm sido explicadas pelo estigma, racismo, inadequação dos serviços e ouros aspectos estruturais ^6^^,^^22^ .

O estudo PrEP1519 foi desenvolvido como resposta a esse contexto, em 2018. Trata-se de ensaio demonstrativo, com desenho longitudinal, para analisar aceitabilidade, factibilidade e efetividade da PrEP para adolescentes de 15 e 19 anos, homens que fazem sexo com homens (HSH), travestis e mulheres trans, nas cidades de Belo Horizonte, Salvador e São Paulo ^23^ . Este estudo, patrocinado pela Unitaid e pelo Ministério da Saúde, buscou articular diferentes abordagens para promover o acesso e permanência em PrEP, como o início da profilaxia no mesmo dia da procura do serviço ^24^ , a criação de demanda por meio de estratégias virtuais ^25^ , incluindo a criação de uma chatbot ^26^ , e presenciais com a participação de educadores de pares, e estratégias de vinculação desenvolvida por navegadores/vinculadores do cuidado e profissionais de saúde ^27^ .

Com essa iniciativa, procurou-se suprir uma ausência expressiva de estudos voltados a adolescentes e o uso de PrEP, que dificultava respostas mais eficientes a essa população, como a não oferta de PrEP no SUS a partir dos 15 anos. Isso passou a ocorrer em 2022, tendo como um dos subsídios os resultados desse estudo.

O presente suplemento reúne um conjunto de conhecimentos produzidos no contexto do estudo PrEP1519, no sentido de melhor compreender as nuances, barreiras e oportunidades para prover o acesso e a permanência de adolescentes em PrEP e no cuidado relacionado ao HIV.

Nesse sentido, o primeiro conjunto de artigos procura elucidar a ocorrência do HIV e das hepatites em adolescentes. Zebalos et al. analisaram os casos de infecção por HIV diagnosticados nas consultas de triagem para PrEP e, utilizando testes laboratoriais de recência, identificaram quais eram as infecções ocorridas recentemente e mostraram a existência de uma taxa expressiva de incidência do HIV em adolescentes.

Também analisando os adolescentes que procuraram os serviços para iniciar PrEP, Santos et al. analisaram biomarcadores para investigar a prevalência de hepatites A, B e C nessa população. Os autores identificaram uma alta carga dessas doenças em uma população que está numa fase inicial das relações sexuais, assim como mostraram que cerca de um quarto dos adolescentes eram não-reagentes para anti-HBc e reagentes para anti-HBs, permitindo melhor conhecer as repercussões das estratégias de imunização nessa população.

Spadacio et al. discutem o potencial da análise temática com sensibilidade interseccional em estudos qualitativos em saúde. Para isso, utilizam como “case” um estudo sobre o continuum de PrEP e examinam como o cruzamento das categorias de diferenciação sociais, analisadas no interior dos temas delineados na análise temática, permite identificar como aspectos estruturais – classe social, gênero, sexualidade e raça/cor da pele – se articulam para produzir diferentes experiências e percepções de desvantagens e privilégios em relação à PrEP.

Dois outros trabalhos examinam dimensões envolvidas na experiência de viver com HIV em adolescentes. Guarnieri et al. analisaram histórias de estigmatização e discriminação e mostraram como as representações sociais do HIV incidem na experiência diagnóstica e no cuidado. Duarte et al. examinam como a PrEP e a carga viral indetectável=intransmissível (I=I) têm produzido tensões e ambivalências nos contextos de encontros afetivo-sexuais de jovens HSH. Mostram, também, como a assimilação dessas tecnologias de saúde constitui importante parte do aprendizado sobre viver com HIV, realçando como a confiança e a maior abertura para novas interações coexistem com dúvidas e ponderações morais. Os trabalhos tornam mais evidente o papel central das políticas públicas na mitigação do estigma e da discriminação, bem como no amplo alcance das informações sobre as tecnologias de prevenção e tratamento do HIV.

O estudo de Rosário et al. procurou conhecer a prevalência de sexo anal sem preservativo entre adolescentes; e mostrou sua associação com a ocorrência de outras práticas que aumentam o risco de infecção, como ter a primeira relação sexual sem preservativo, usar substâncias psicoativas e o sexo transacional. Deixou, assim, evidente a multidimensionalidade de fatores envolvidos nas práticas sexuais desprotegidas e reforçou a importância de políticas de saúde sexual e de serviços qualificados e integrados para esta população.

Grangeiro et al. investigaram se a oferta de PrEP na comunidade permitia reduzir barreiras e propiciar que adolescentes mais vulneráveis tivessem um acesso mais oportuno à PrEP. Para isso, com equipes extramuros, implantaram a oferta em duas organizações comunitárias e comparam o perfil dos adolescentes atendidos nessas organizações com a atendida em um serviço convencional de saúde. Os resultados evidenciaram que uma oferta de PrEP simplificada e próxima da comunidade contribuiu para reduzir inequidades e ampliar o acesso a profilaxia.

Um conjunto formado por três artigos empregou a pesquisa qualitativa para refletir sobre a oferta e o uso da PrEP. Urbano et al. usaram o conceito de cuidado para examinar as percepções e as práticas de profissionais de saúde sobre a oferta da PrEP. Os resultados mostraram que a construção de vínculo de confiança, escuta sensível e solidária, o reconhecimento da singularidade e a vulnerabilidades de adolescentes são essenciais para apoiar o desenvolvimento da autonomia na prevenção do HIV. Destacaram, também, concepções adultocêntricas, que figuram como pontos de tensão entre o êxito técnico e o sucesso prático.

No segundo artigo, Oliveira et al. utilizam uma perspectiva interseccional para examinar como os sistemas de opressão configuram o continuum do cuidado da PrEP de jovens HSH. Isso permitiu evidenciar a centralidade do racismo para adolescentes negros construírem um conhecimento menos técnico sobre PrEP do que brancos. Apesar de profissionais reconhecerem tais opressões, poucos manejam esse aspecto para transformar a prática do cuidado.

Com foco nas repercussões da pandemia da covid-19 em serviços de PrEP, o trabalho de Unsain et al. examinou como mulheres transexuais, travestis e pessoas não binárias ou de gênero fluido (MTrT+) ressignificaram suas lutas, cotidianos e formas de cuidado. Para essas populações, as mudanças nos serviços de PrEP foram positivas, por diminuírem o tempo de permanência ou reduzirem a frequência ao serviço de saúde, pela substituição das consultas por contatos virtuais e o recebimento de medicação em casa. As autoras discutem a centralidade da manutenção da territorialização da PrEP durante a pandemia, a partir da construção de uma relação com os serviços e os profissionais como suficientemente eficientes em situações habituais e de crise.

O trabalho de Deus et al. se debruça sobre as percepções dos adolescentes acerca de um novo esquema de uso de PrEP, o sob demanda, e destaca a relação entre o desconhecimento desse esquema e as preocupações relacionadas à efetividade e à segurança. Mostraram, ainda, uma tensão entre as vantagens desse esquema de PrEP para uma maior autonomia na gestão da prevenção e a redução do uso de medicamentos, com preocupações em relação à imprevisibilidade das relações sexuais e as dificuldades em utilizar corretamente as dosagens.

Ao examinar o impacto da pandemia da covid-19 no acesso ao autoteste anti-HIV, Magno et al. estimaram fatores associados à solicitação do autoteste no período anterior e durante a pandemia. Entre os principais achados está a associação do uso do autoteste com situações de maior risco no período anterior à pandemia e uma diminuição desse uso durante a pandemia, especialmente entre aqueles com parcerias estáveis. Ao realçarem as mudanças nas dinâmicas sexuais de adolescentes e sua relação com a testagem durante a pandemia, os resultados contribuem para informar estratégias de otimização da oferta do autoteste.

Esperamos que o conjunto dos trabalhos deste suplemento, ao conjugar conhecimentos mais aprofundados sobre a oferta de diferentes modalidades de PrEP, as práticas profissionais, a diversificação de modelos de serviço e, sobretudo, as necessidades de adolescentes, contribua para disseminar e subsidiar práticas mais efetivas e inovadoras na prestação de serviços, no acesso à PrEP, diagnóstico oportuno das infecções e início da terapia antirretroviral. Ressaltamos, também, nossa convicção de que a produção de conhecimento requer adensamento crítico e transformações efetivas para promover e sustentar a participação das comunidades nessa construção de saberes.
