# Supplementary material for: New horizons in sexual health: exploring PrEP and HIV incidence in adolescents
Source: Rev Saude Publica. 2024 Oct 11;58(Suppl 1):2s. doi: 10.11606/s1518-8787.2024058supl1ap (PMC11573370; doi:10.11606/s1518-8787.2024058supl1ap)
Supplement: Translation

# Translation: Novos horizontes na saúde sexual: explorando a PrEP e a incidência de HIV em adolescentes

**Authors:** Alexandre Grangeiro, Eliane Miura Zucchi, Laio Magno, Unai Tupinambás, Dirceu Bartolomeu Greco, Inês Dourado

**Affiliations:** I Universidade de São Paulo. Faculdade de Medicina. São Paulo, SP, Brasil Universidade de São Paulo Faculdade de Medicina SP São Paulo Brasil; II Universidade Católica de Santos. Programa de Pós-Graduação em Saúde Coletiva. Santos, SP, Brasil Universidade Católica de Santos Programa de Pós-Graduação em Saúde Coletiva SP Santos Brasil; III Universidade do Estado da Bahia. Departamento de Ciências da Vida. Salvador, BA, Brasil Universidade do Estado da Bahia Departamento de Ciências da Vida BA Salvador Brasil; IV Universidade Federal de Minas Gerais. Faculdade de Medicina. Belo Horizonte, MG, Brasil Universidade Federal de Minas Gerais Faculdade de Medicina MG Belo Horizonte Brasil; V Universidade Federal da Bahia. Instituto de Saúde Coletiva. Salvador, BA, Brasil Universidade Federal da Bahia Instituto de Saúde Coletiva BA Salvador Brasil

## PRESENTATION

The United Nations goal of eliminating HIV as a public health problem by 2030 is feasible from a theoretical and scientific point of view ^1^ . In fact, several studies have shown that the timely treatment of people living with HIV and preventive actions with the available health technologies is highly effective in significantly reducing HIV incidence and mortality ^2^ . However, achieving this goal has faced major challenges, such as inequalities, stigma, insufficient funding, deficits in the structure of services and intersectoral articulation, and relatively low engagement of communities and policymakers ^1^^,^^3^^–^^6^ .

In view of this, the United Nations Programme on HIV/AIDS has drawn attention to the fact that the countries that have progressed the most toward eliminating HIV have promoted information; focused on actions that most impact the epidemic, mobilization, and social participation; and properly articulated their response and actors ^1^ . This occurs in different contexts, such as in African countries heavily hit by the epidemic ^7^ and in those with higher income ^8^ .

On the other hand, the profile of the HIV epidemic in Brazil has significantly changed as it combines a new wave of increased incidence ^9^^,^^10^ , the consolidation of regional sub-epidemics marked by social inequalities ^11^^–^^13^ , and a growing incidence among adolescents and young adults, especially among cisgender men who have sex with men, *travestis* , and transgender women ^9^^,^^14^ .

A combination of life cycle factors ^14^ can explain the increase in the epidemic in adolescents, such as experimentation with sexual practices, the need to handle stigma related to sexual orientation and gender identity from an early age, and a generational change marked by the perception of the decreased severity of HIV and new ways of exercising sexuality. This has led to a kind of vicious cycle that increases the frequency of sexual onset without the use of condoms and of unprotected sex ^15^ , especially in adolescents under greater social vulnerability ^16^ .

An opportunity to reverse this scenario has been pre-sexual exposure prophylaxis (PrEP), which, combined with other preventive methods, is a highly effective option ^17^ for populations at higher risk of infection, especially those who are unwilling or unable to consistently use condoms. Increased population PrEP use rates are associated with decreased incidence ^4^^,^^8^^,^^18^ , which tends to be even more accentuated with the introduction of new long-acting prophylactic regimens that offer greater convenience and promise to reduce non-adherence and use interruption ^2^ . One such example refer to the results of a clinical trial, called Purpose 1, carried out with more than 2000 cisgender women, which used an injectable drug applied every six months and found no HIV infections throughout the evaluated period ^19^ .

Despite these promising results, PrEP use has been marked by access inequalities, focusing primarily on populations and regions with higher socioeconomic status ^2^^,^^6^^,^^20^ . In Brazil, for example, this inequality affects the most socially vulnerable populations the most; so much so that cis women, including sex workers, are 10 times less likely to use PrEP than cis men. This difference is twice as large between Black and White people and between those aged 18 to 24 years and 30 to 39 years ^21^ . These inequalities have been explained by stigma, racism, inadequacy of services, and other structural aspects ^6^^,^^22^ .

The PrEP1519 study was developed in 2018 as a response to this context. This longitudinal demonstrative trial aimed to analyze PrEP acceptability, feasibility, and effectiveness in adolescents aged from 15 and 19 years, men who have sex with men (MSM), *travestis* , and trans women in the municipalities of Belo Horizonte, Salvador, and São Paulo ^23^ . Sponsored by Unitaid and the Ministry of Health, this study sought to articulate different approaches to promote access and adherence to PrEP, such as starting PrEP on the first consultation ^24^ , fostering demand by virtual strategies ^25^ , including creating a chatbot ^26^ , promoting in-person with the participation of peer educators, and developing bonding strategies with care navigators/linkers and healthcare providers ^27^ .

This initiative sought to make up for the significant lack of studies in adolescents and the use of PrEP, which hindered more efficient responses to this population, such as the non-provision of PrEP in SUS from the age of 15 years onward. Since 2022, the study subsidized such initiatives as one of its results.

This supplement gathers a set of knowledge produced in the PrEP1519 study to better understand the nuances, barriers, and opportunities to provide adolescents with access to PrEP and HIV-related care and promote their adherence to it.

Thus, the first set of articles seeks to explain the occurrence of HIV and hepatitis in adolescents. Zebalos et al. analyzed cases of HIV infection diagnosed in PrEP screening consultations and, using laboratory tests, found which infections had occurred recently, showing a significant rate of HIV incidence in adolescents.

Also analyzing adolescents who sought services to begin PrEP, Santos et al. analyzed biomarkers to investigate the prevalence of hepatitis A, B, and C in this population, finding a high burden of these diseases in a population on the onset of sexual activity as about a quarter of these adolescents were non-reactive for anti-HBc and reactive for anti-HBs, thus better evaluating the repercussions of immunization strategies for them.

Spadacio et al. discussed the potential of thematic analysis with intersectional sensitivity in qualitative health studies. To do so, they use as a “case” a study on the PrEP continuum and examine how the intersection of the categories of social differentiation—analyzed within the themes outlined in the thematic analysis—can find how structural aspects—social class, gender, sexuality, and race/skin color—are articulated to produce different experiences and perceptions of disadvantages and privileges toward PrEP.

Moreover, two studies examine dimensions involved in the experience of living with HIV in adolescents. Guarnieri et al. analyzed stories of stigmatization and discrimination and showed how social representations of HIV affect the diagnostic experience and care. Duarte et al. examined how PrEP and the undetectable=untransmittable viral load have produced tensions and ambivalences in young MSM’s affective-sexual encounters. They showed how assimilating these health technologies constitutes an important part of learning about living with HIV, highlighting how trust and greater openness to new interactions coexist with doubts and moral considerations. These studies further evince the central role of public policies in mitigating stigma and discrimination and the wide reach of information on HIV prevention and treatment technologies.

Rosário et al. sought to determine the prevalence of condomless anal sex in adolescents, showing its association with other practices that increase the risk of infection, such as having the first sexual intercourse without a condom, using psychoactive substances, and engaging in transactional sex, thus evincing the multidimensionality of factors in unprotected sexual practices and reinforcing the importance of sexual health policies and well-trained and integrated services for this population.

Grangeiro et al. investigated whether providing PrEP in a community could reduce barriers and provide more vulnerable adolescents with more timely access to PrEP. For this they implemented PrEP with extramural teams in two community organizations and compared the profile of adolescents treated in these organizations with that of those who received care in a conventional health service. Results showed that a simplified and community-friendly PrEP offer contributed to reducing inequities and expanding access to prophylaxis.

A set of three articles used qualitative research to reflect on the supply and use of PrEP. Urbano et al. used the concept of care to examine healthcare providers’ perceptions and practices about PrEP offer. Results showed that building a bond of trust, offering sensitive and supportive listening, and recognizing adolescents’ uniqueness and vulnerabilities are essential to support the development of autonomy in HIV prevention. They also highlighted adultcentric conceptions that figure as points of tension between technical success and practical success.

In the second article, Oliveira et al. used an intersectional perspective to examine how systems of oppression shape the continuum of PrEP care for young MSM. This made it possible to highlight the centrality of racism for Black adolescents to build less technical knowledge about PrEP than White adolescents. Although professionals recognize such oppressions, few manage this aspect to transform the practice of care.

Focusing on the repercussions of the COVID-19 pandemic on PrEP services, Unsain et al. examined how transgender women, transvestites, and non-binary or gender-fluid (MTrT+) women have reframed their struggles, daily lives, and forms of care. These populations deemed the changes in PrEP services as positive as they shortened length of stay or reduced attendance at health services, replaced appointments with virtual contacts, and delivered medication at home. The authors discussed the centrality of maintaining the territorialization of PrEP during the pandemic by building a sufficiently efficient relationship with services and professionals in usual and crisis situations.

Deus et al. focused on adolescents’ perceptions of a new PrEP on-demand regimen, highlighting the relationship between the lack of knowledge of this regimen and concerns related to its effectiveness and safety. They also evinced the tension between the advantages of this PrEP regimen toward greater autonomy in managing prevention and reducing medication use with concerns about the unpredictability of sexual relations and the difficulties in correct dosages.

Examining the impact of the COVID-19 pandemic on access to HIV self-testing, Magno et al. estimated factors associated with the request for self-testing in the period before and during the pandemic. Their main findings include the association of self-testing with higher risk before the pandemic and a decrease in its use during it, especially in those with stable partners. By highlighting the changes in the sexual dynamics of adolescents and their relationship with testing during the pandemic, results contribute to inform strategies to optimize the offer of self-testing.

We hope that the set of studies in this supplement, by combining more in-depth knowledge about the provision of different PrEP modalities, professional practices, service model diversification, and, above all, the needs of adolescents, will contribute to disseminate and subsidize more effective and innovative practices in service provision, access to PrEP, timely diagnosis of infections, and initiation of antiretroviral therapy. We also stress our conviction that producing knowledge requires critical densification and effective transformations to promote and sustain the participation of communities in this construction of knowledge.
